# The emerging threat antifungal-resistant *Candida tropicalis* in humans, animals, and environment

**DOI:** 10.3389/ffunb.2022.957021

**Published:** 2022-08-15

**Authors:** Ricardo Lima, Felipe C. Ribeiro, Arnaldo L. Colombo, Joăo N. de Almeida

**Affiliations:** ^1^ Special Mycology Laboratory, Escola Paulista de Medicina, Universidade Federal de São Paulo, São Paulo, Brazil; ^2^ Clinical Laboratory, Hospital Israelita Albert Einstein, São Paulo, Brazil

**Keywords:** *Candida tropicalis*, animals, humans, environment, resistance

## Abstract

Antifungal resistance in humans, animals, and the environment is an emerging problem. Among the different fungal species that can develop resistance, *Candida tropicalis* is ubiquitous and causes infections in animals and humans. In Asia and some Latin American countries, *C. tropicalis* is among the most common species related to candidemia, and mortality rates are usually above 40%. Fluconazole resistance is especially reported in Asian countries and clonal spread in humans and the environment has been investigated in some studies. In Brazil, high rates of azole resistance have been found in animals and the environment. Multidrug resistance is still rare, but recent reports of clinical multidrug-resistant isolates are worrisome. The molecular apparatus of antifungal resistance has been majorly investigated in clinical *C. tropicalis* isolates, revealing that this species can develop resistance through the conjunction of different adaptative mechanisms. In this review article, we summarize the main findings regarding antifungal resistance and *Candida tropicalis* through an “One Health” approach.

## Introduction

The yeast *Candida tropicalis* is a member of the kingdom *Fungi*, division *Ascomycota*, order *Saccharomycetales*. They are diploid dimorphic yeasts, showing ellipsoidal budding cells or as a pseudomycelium and can form true hyphae (Ann Chai et al., 2010). On Sabouraud dextrose agar *C. tropicalis* grows as cream-colored colonies with a slightly mycelial border, however, it is impossible to differentiate it from other *Candida* species. On CHROMagar™ Candida (CHROMagar Company Ltd), after 24-72 hours, colonies appear a metallic blue coloration, and that can be used as presumptive identification (Ann Chai et al., 2010; Silva et al., 2012).

Molecular data shows that *C. tropicalis* belong to the CUG-Ser 1 clade, meaning that the codon CUG is translated to serine instead of leucine (Krassowski et al., 2018). The diploid genome of *C. tropicalis* has about 14.5 Mb, and the species can mate *via* a parasexual cycle (Butler et al., 2009; Porman et al., 2011).

The pathogenicity of *C. tropicalis* is mediated by several virulence factors including extracellular secreted enzymes (phospholipase, hemolysin, coagulase, and proteinase), biofilm formation, and antifungal resistance. These extracellular enzymes contribute to *C. tropicalis* virulence, and, they have been reported to disrupt the host tissue, increasing the dissemination and evasion of macrophages after yeast cells phagocytosis (Togni et al., 1996; Zaugg et al., 2001; Deorukhkar et al., 2014; Staniszewska, 2020; Sasani et al., 2021). *C. tropicalis* is a greater biofilm producer in the *Candida* genus (Kumari et al., 2018), and mature biofilms are composed of a dense network of blastoconidia, containing an adhesive basal layer with cells that can form filaments with a large amount of extracellular matrix (Al-Fattani and Douglas, 2006; Soll, 2014; Araújo et al., 2017; Zheng et al., 2017; Dominguez et al., 2018). The extracellular matrix is composed of carbohydrates, proteins, phosphorus, uronic acid, and hexosamine, the major component in the matrix biofilm (Al-Fattani and Douglas, 2006).

Ubiquitous, *C. tropicalis* and has been recovered from different environmental sources. In Taiwan, the species has been retrieved from agricultural fields, forest soil, petroleum-contaminated, and sludge soil (Yang et al., 2012). In tropical China, *C. tropicalis* is the most frequent yeast found in the environment (Liu et al., 2022). One study that collected 968 environmental samples from soil, freshwater, and seawater from eight regions in tropical China showed 21% positivity for *C. tropicalis* (Liu et al., 2022). Brazilian researchers have shown that *C. tropicalis* can be found in the Amazon forest soil (Mok et al., 1984), agricultural fields (Sidrim et al., 2021), rivers and lakes (Medeiros et al., 2008), sand beaches (Zuza-Alves et al., 2019), sugar cane bagasse (Lara et al., 2014), coconut water (Maciel et al., 2013). It has also been recovered from soil samples collected in the USA and Ireland (O’Brien et al., 2021).

Microbiota analysis of terrestrial and marine mammals, as well as birds, crustaceans, and insects, have also revealed the presence of *C. tropicalis* (Takahashi et al., 2010; Hamad et al., 2014; Cordeiro et al., 2015). In humans, *C. tropicalis* can be found on the skin (Kam and Xu, 2002), nails (Kam and Xu, 2002), oral mucosa (Cui et al., 2015), gut (Hager and Ghannoum, 2017), and vagina (Barousse et al., 2004). Of note, patients with Crohn’s disease have increased levels of *C. tropicalis* in the gut compared to healthy individuals (Hager and Ghannoum, 2017).

Beyond colonization, *C. tropicalis* has been related to different infections in a wide range of animals, from gastrointestinal disease in sows (Zhai et al., 2021), bovine mastitis (Seker, 2010), oral mucositis in vultures exposed to antibiotics from livestock (Pitarch et al., 2020), urinary tract infection in dogs and cats (Ozawa et al., 2005), to genital tract infection in mares (Shokri et al., 2010). In humans, *C. tropicalis* has been related to superficial mycosis like onychomycosis (Arrua et al., 2015), otomycosis (Sabz et al., 2019), oral (Salehi et al., 2020) or skin candidiasis (Sanchez et al., 2021), keratitis (Rosa et al., 1994), and genital tract infections (Mushi et al., 2019). Regarding the invasive infections, *C. tropicalis* is an important agent of candidemia, ranking as the most common species in medical centers in India, Pakistan (Wang et al., 2016), Thailand (Ngamchokwathana et al., 2021), Algeria (Megri et al., 2020), and Tunisia (Sellami et al., 2011), and as the second most common *Candida* species in medical centers from, Brazil (Colombo et al., 2006; Agnelli et al., 2022), Colombia (Cortés et al., 2013), Venezuela (Dolande Franco et al., 2008), Mexico (Corzo-Leon et al., 2014), China (Liu et al., 2021), Singapore (Teo et al., 2017). Among countries in North hemisphere, cancer patients have a higher risk to be infected with *C. tropicalis* and fungemia by this species is associated to mortality rates that are usually above 40% (Kontoyiannis et al., 2001; Muñoz et al., 2011). In Asia and Latin America, *C. tropicalis* has been documented as a common agent of candidemia not only in cancer patients but also in adult and child critically ill patients (Nucci and Colombo, 2007; Agnelli et al., 2022).

## The burden of antifungal-resistant *Candida tropicalis* in humans, animals, and the environment

Recent evidence points out that there is an increasing prevalence of fluconazole and multidrug resistance among *C. tropicalis* recovered from humans (Favarello et al., 2021; Wang et al., 2021). In China, some centers report a prevalence of 20-50% of fluconazole non-susceptibility among *C. tropicalis* clinical isolates (Fan et al., 2019; Wang et al., 2020; Wang et al., 2021), while in Algeria and Japan, reports show that more than 30% of *C. tropicalis* bloodstream isolates are fluconazole-resistant (Megri et al., 2020; Khalifa et al., 2022). In Turkey, a recent multicentric study showed that over 10% of the bloodstream isolates collected from 2017 to 2019 are resistant to fluconazole (Arastehfar et al., 2020b).

The burden of antifungal resistance in *C. tropicalis* has been well estimated in India (Mathews et al., 2001; Chakrabarti et al., 2015). Based on a multicentric study (Chakrabarti et al., 2015), a burden of 6.51 candidemia cases per 1000 ICU admissions were estimated, of which 41.6% (n=382) were *C. tropicalis* and resistance rates against fluconazole was 2.6% (n=10). In Brazil, the incidence of candidemia in Brazil is over 2 cases per 1000 admissions (Nucci et al., 2013). Taking into account a prevalence of 20% of the *C. tropicalis* in blood cultures (da Matta et al., 2017), and that approximately 4% of the *C. tropicalis* bloodstream isolates are fluconazole-resistant in the country (Favarello et al., 2021), one can estimate that for every 100000 admissions there will be at least one case of ARCT candidemia episode in Brazil.

The emergence of fluconazole resistance leads to echinocandin or amphotericin exposure and multidrug resistance is a future concern with *C. tropicalis* and human health. Despite its low prevalence (<1%), azole and echinocandin resistant *C. tropicalis* clinical isolates have been reported in the USA, in cancer patients (Garcia-Effron et al., 2008; Kofteridis et al., 2010; Sfeir et al., 2020), and Taiwan (Chen et al., 2019) and India (Chakrabarti et al., 2015). Amphotericin B resistance is also possible (Yang et al., 2004; Forastiero et al., 2013), although it is rarely reported in the world.

The burden of antifungal resistant *C. tropicalis* in animals is less estimated than it is in humans. However, the high prevalence of ARCT in animals has been pointed out in some studies (Khalifa et al., 2022). In Japan, 4% to 75% of *C. tropicalis* recovered from aquarium dolphins were fluconazole-resistant (Takahashi et al., 2010; Khalifa et al., 2022). In Brazil, over 50% of the *C. tropicalis* isolates from the microbiota of healthy animals (goat, sheep, rheas, psittacines, horses, sirenians, shrimp, tortoises, sea turtles) were also considered fluconazole-resistant (Brilhante et al., 2015; Cordeiro et al., 2015). In India, one study showed that *C. tropicalis* from health poultry microbiome had high fluconazole minimal inhibitory concentrations [MICs (4-16mg/L)] (Subramanya et al., 2017).

Rates of azole resistance in environmental isolates are still poorly investigated. In one Brazilian study, a high level of azole resistance in environmental *C. tropicalis* isolates was documented (Zuza-Alves et al., 2016a). Briefly, the study was carried out at the Northeastern coastal area from Brazil, and *C. tropicalis* isolates from sandy beaches showed 25% of azole resistance (Zuza-Alves et al., 2016a). A robust study that analyzed the prevalence of yeasts and antifungal resistance among environmental samples in tropical China revealed that over 21% of the *C. tropicalis* isolates were fluconazole-resistant (Liu et al., 2022).

## Known antifungal tolerance and resistance mechanisms

*C. tropicalis* is known to have an effective response against osmotic stress being able to tolerate high salt concentrations. This species can grow in the presence of 10-15% sodium chloride and it was isolated in the Dead Sea and Amazonian forest in a hypersaline environment (Tokuoka, 1993; Ribeiro Bastos et al., 2000; Butinar et al., 2005). Its ability to tolerate an environment with high osmotic pressure is related to the activation of transporters that promote a rapid efflux of ions by efflux pumps (García et al., 1997; Zuza-Alves et al., 2017). A study has demonstrated the osmotolerance of *C. tropicalis* isolates obtained from the coastal environment in Brazil and its relation to virulence expression *in vitro* (Zuza-Alves et al., 2016b). The authors suggested that the presence of these yeasts in coastal environments may have led to the overexpression of efflux pumps, which may explain the high MICs values found on several drugs (azoles and amphotericin B). Another relevant biologic characteristic of *C. tropicalis* is its ability to tolerate and degrade phenol (Páca et al., 2005; Wang et al., 2011). Indeed, phenol is an aromatic compound applied in the production of pesticides, antiseptics, slimicides, and medicinal preparations (Mishra and Kumar, 2019), and is considered a marker of environmental pollution (Montaño et al., 2013). Recently, a study showed that *C. tropicalis* exposed to phenol compounds accumulated intracellular fatty acids and cell wall modifications that promote stress resistance. Moreover, the study also showed that phenol exposure leads to upregulation of efflux mechanisms correlated with azole resistance (Wang et al., 2011).

Antifungal tolerance precedes resistance and it has been investigated in clinical *C. tropicalis* strains. The calcineurin signaling pathway is activated under stress and turns *C. tropicalis* tolerant to azoles and echinocandins (Chen et al., 2014). The protein kinase A pathway and its catalytic subunits Tpk1 have also been associated with cell wall integrity and stress tolerance (Lin et al., 2018). The trailing phenomenon or residual growth above the minimal inhibitory concentration has been considered by some authors as a phenotypic expression of drug tolerance (Arastehfar et al., 2020b) or a less-susceptible phenotype (Choi et al., 2016a). Indeed, trailing-producing *C. tropicalis* strains have higher expression of efflux pumps compared to the azole wildtype ones (Astvad et al., 2018). Despite being a different population, tolerant or trailing-producing *C. tropicalis* strains (growth inhibition >50%) are not related to treatment failure. A study demonstrated that only microorganisms showing fluconazole growth inhibition that fulfill the definition of resistance (growth inhibition <50% starting from the MIC of 4mg/L) had treatment failure in an *in vivo* murine model of disseminated candidiasis (Astvad et al., 2018). Moreover, a multicentric study conducted in Spain showed that candidemia by trailing-producing *Candida* spp. was not associated with azole treatment failure (Rueda et al., 2017). Other authors (Colombo-Judith Berman) have found a correlation between fluconazole-tolerant *Candida albicans* and persistent candidemia in patients treated with fluconazole (Rosenberg et al., 2018).

Among the main factors in the development of fluconazole resistance, mutations in the ergosterol biosynthesis pathway genes are well characterized in *C. tropicalis*. The enzyme lanosterol 14 α-demethylase encoded by the *ERG11* gene is the main target for azole antifungals (Cowen et al., 2014; Maubon et al., 2014; Sanglard, 2016; Robbins et al., 2017; Jordá and Puig, 2020). At least 31 mutations in *ERG11* may be correlated to fluconazole resistance in *C. tropicalis* (**Table 1**), and the most common mutations are Y132F and S154F (Morio et al., 2010; Jiang et al., 2013; Cowen et al., 2014; Perlin et al., 2017; Robbins et al., 2017; Ksiezopolska and Gabaldón, 2018; Fan et al., 2019; Teo et al., 2019; Zhang et al., 2019; Castanheira et al., 2020).

**Table 1:** Molecular mechanisms of resistance to fluconazole or echinocandins in *C. tropicalis* from invasive fungal infections.

Mutations		Reference
**ERG11- Coding ERG11 protein (lanosterol 14-α demethylase in *Candida*)**		
ERG11	Y132F	(Forastiero et al., 2013; Jiang et al., 2013; Tan et al., 2015; Chew et al., 2017; Jin et al., 2018; Chew et al., 2019b; Fan et al., 2019; Teo et al., 2019; Zhang et al., 2019; Arastehfar et al., 2020b; Castanheira et al., 2020; Chen et al., 2021; Chew et al., 2021)
	K143R/X	(Xisto et al., 2017)
	R245K	(Arastehfar et al., 2020b)
	Y221F	(Arastehfar et al., 2020b)
	K344N/T	(Arastehfar et al., 2020b)
	V326M	(Arastehfar et al., 2020b)
	Y257H	(Chew et al., 2019b; Fan et al., 2019)
	V125A	(Fan et al., 2019)
	F145L	(Teo et al., 2019)
	S154F	(Jiang et al., 2013; Chew et al., 2019b; Teo et al., 2019; Arastehfar et al., 2020b; Castanheira et al., 2020; Chen et al., 2021)
	T225C	(Álvarez-Pérez et al., 2016b)
	G264A	(Álvarez-Pérez et al., 2016b)
	G1362A	(Álvarez-Pérez et al., 2016b)
	T1554C	(Álvarez-Pérez et al., 2016b)
	A427M	(Xisto et al., 2017)
	G464S/D	(Forastiero et al., 2013; Choi et al., 2016b; Fan et al., 2019)
	V362M/I	(Choi et al., 2016b; Arastehfar et al., 2020b)
	T225Y	(Xisto et al., 2017)
	G264R	(Xisto et al., 2017)
	T342Y/C	(Xisto et al., 2017)
	A428G	(Xisto et al., 2017)
	Y132C	(You et al., 2017a)
	T224C	(Benedetti et al., 2019)
	G263A	(Benedetti et al., 2019)
	D454N	(Chen et al., 2021)
	Y132F + S154F	(Arastehfar et al., 2020b; Chew et al., 2021)
**ERG3 – Coding ERG3 protein (enzyme sterol Δ^5,6^ desaturase in *Candida*)**		
ERG3p	ERG3 - 2-bp insertion in positions 1130 and 1131	(Álvarez-Pérez et al., 2016b)
	**S113G**	(Forastiero et al., 2013)
**UPC2 – Coding a zinc cluster transcription factor of ERG genes in *Candida***		
UPC2p	A251T	(Choi et al., 2016b)
	Q289L	(Choi et al., 2016b)
	A297S	(Choi et al., 2016b)
	T393I	(Choi et al., 2016b)
	A251T	(Choi et al., 2016b)
	G392E	(Choi et al., 2016b; Jiang et al., 2016, 3)
	Q289L	(Choi et al., 2016b)
	L343F	(Choi et al., 2016b)
	S187L	(Choi et al., 2016b)
	T241A	(Arastehfar et al., 2020a)
	Q340H	(Arastehfar et al., 2020a)
	T381S	(Arastehfar et al., 2020a)
	Promoter region in *C. tropicalis*, -118T-G and -155G-A	(Jiang et al., 2016, 2)
**MRR1 – multidrug resistance regulator 1 in *Candida***		
MRR1p	T255P	(Arastehfar et al., 2020a)
	T647S	(Arastehfar et al., 2020a)
**TAC1 (transcriptional activator of C *DR* genes) is a zinc-cluster transcription in *Candida***		
TAC1	N164I	(Arastehfar et al., 2020a)
**Other mutations related to resistance**		
MDR1p Multi-Drug Resistance 1	E133D	(Castanheira et al., 2020)
	V76A	(Castanheira et al., 2020)
	A189V	(Castanheira et al., 2020)
	P448L	(Chew et al., 2019b)
CDR2p ATP binding cassette (ABC) transporters	CDR2 K427_ stop codon	(Castanheira et al., 2020)
**Detection of genes overexpressed by qPCR**		
CDR1		(Fan et al., 2019; Teo et al., 2019)
MDR1		(Kanoshiki et al., 2015; You et al., 2017a; Jin et al., 2018; Fan et al., 2019; Teo et al., 2019; Khalifa et al., 2022)
UPC2		(Jiang et al., 2016, 2; Wang et al., 2021; Khalifa et al., 2022)
ERG11		(Jiang et al., 2013; Kanoshiki et al., 2015; Jin et al., 2018; Fan et al., 2019; Teo et al., 2019; Wang et al., 2021; Khalifa et al., 2022)
CDR2		(Khalifa et al., 2022)
CDR3		(Khalifa et al., 2022)
TAC1		(Khalifa et al., 2022)
HMG		(Khalifa et al., 2022)
**FKS1 1,3-beta-glucan synthase componente in *Candida***		
Hot Spot1	F650S	(Castanheira et al., 2020)
	S654P	(Castanheira et al., 2020; Sfeir et al., 2020)
	S645P	(Grosset et al., 2016; Khan et al., 2018b; Chew et al., 2019b; Díaz-García et al., 2021)
	S80S/P	(Jensen et al., 2013; Sfeir et al., 2020)
	R656G/R	(Díaz-García et al., 2021)
	S80P	(Xiao et al., 2018b; Sfeir et al., 2020)
	D648V	(Chew et al., 2019b)
	F641S/L	(Chew et al., 2019b; Sfeir et al., 2020)
Hot Spot 2	M1235I	(Chew et al., 2019b)

The increased expression of genes related to the ergosterol biosynthesis process constitutes another mechanism of resistance to triazoles in *C. tropicalis*. These mechanisms are associated with the overexpression of the transcription factor UPC2, or with the formation of isochromosomes (Jiang et al., 2013; Cowen et al., 2014; Kanoshiki et al., 2015; Choi et al., 2016b; Perlin et al., 2017; Robbins et al., 2017; Jin et al., 2018; Fan et al., 2019; Teo et al., 2019).

Activation of efflux pumps is also described as an important mechanism of resistance to azole antifungal agents and in *Candida* spp. two different drug efflux systems have been associated with resistance to triazoles: the ATP-Binding Cassette (ABC) superfamily of proteins, and the Major Facilitator Superfamily (MFS) carriers (Sanglard, 2016; Perlin et al., 2017; Robbins et al., 2017; You et al., 2017a, 1). Proteins belonging to the ABC superfamily are primary transporters that hydrolyze ATP molecules and are essential to the transport of substrates. In *C. tropicalis*, the transporters named *Candida* Drug Resistance 1 and 2 (CDR1 and CDR2) are well characterized as responsible for the increase in resistance to triazoles (**Table 2**) (Cowen et al., 2014; Perlin et al., 2017; Robbins et al., 2017; Khalifa et al., 2022). The second efflux system associated with triazole resistance involves MFS transporters, which transport various substrates using a proton gradient generated in the plasma membrane. Among the described MFS transporters, only the Multi-Drug Resistance 1 (MDR1) gene has been associated with azole resistance in clinical isolates of *C.tropicalis* (**Table 1**) (Cowen et al., 2014; Perlin et al., 2017; Robbins et al., 2017; You et al., 2017a; Jin et al., 2018, 1). Moreover, gain-of-function mutations in transcription factors such as *MMR1* and *TAC1* genes were also described in *C. tropicalis* and can lead to higher expression of drug efflux pumps (**Table 1**).

**Table 2:** Prevalence of *Candida tropicalis* from different Asian countries, niches, and multilocus sequence typing data.

Country	Prevalence in humans	Prevalence of azole resistance in humans	Prevalence of azole resistance in animal source	Prevalence of azole resistance in the environment	Azole-resistant MLST cluster	Main azole-resistant DSTs	Common azole-resistant DST human-environment	Ref.
**China**	**High**	**High**	NI	**High**	CC2*	**DST225, DST525, DST546**	**DST225**	(Wang et al., 2016; Wang et al., 2020; Liu et al., 2022)
**Taiwan**	**High**	**High**	NI	**High**	**CC1, CC3, CC10, CC11**	**DST225, DST506, DST507, DST376**	**DST149, DST225**	(Yang et al., 2012; Lo et al., 2017; Chen et al., 2019)
**Japan**	**Low**	**High**	**Low**	**Low**	No	**DST45, DST134, DST140, DST162, DST378**	No	(Sakamoto et al., 2021; Khalifa et al., 2022)
**Singapore**	**High**	**High**	NI	NI	CC4*	**DST544, DST543**	NI	(Tan et al., 2016; Wang et al., 2020)
**Korea**	**High**	**Low**	NI	NI	NI	NI	NI	(Ko et al., 2019; Kim et al., 2020)
**Thailand**	**High**	**High**	NI	NI	**CC1**	**DST506, DST1101, DST1097**	NI	(Wang et al., 2016; Boonsilp et al., 2021)
**India**	**High**	**Low**	**High**	NI	CC17*	**DST216, DST217**	NI	(Chakrabarti et al., 2015; Wang et al., 2016; Subramanya et al., 2017; Wang et al., 2020)
**Pakistan**	**High**	**Low**	NI	NI	NI	NI	NI	(Farooqi et al., 2013; Wang et al., 2016)

Low prevalence: <5%; Moderate prevalence 5-10%; High prevalence: >10%; NI, not investigated; MLST, multilocus sequence typing; DST, Diploid sequence type; CC, clonal complex. *Genetically close-related clonal complexes.

Like in other *Candida* spp., echinocandin resistance is associated with mutations in the FKS1 gene (β-1, 3-D-glucan synthase, hotspot 1) in C. *tropicalis* (**Table 1**) (Cowen et al., 2014; Robbins et al., 2017; Khan et al., 2019; Pfaller et al., 2019; Castanheira et al., 2020).

Amphotericin B resistance is rare in *C. tropicalis* and only a few studies have tried to explore it. In Spain, a clinical isolate showing azole and amphotericin B cross-resistance had ERG11p (G464D) and ERG3p (S113G) mutations associated with the absence of ergosterol in the cell membrane (Forastiero et al., 2013). Moreover, it also had increased β-D-glucan in the cell wall, tolerance to reactive oxygen species, and stronger production of cytokines by peripheral blood mononuclear cells than amphotericin B- susceptible isolates (Mesa-Arango et al., 2014; Mesa-Arango et al., 2016).

## Reservoirs of antifungal resistance

The human digestive tract is a relevant source of ARCT. Immunocompromised or critically ill patients exposed to antimicrobials and antifungals may have the selection of ARCT in the digestive tract and further develop an infection if the mucosa barrier is damaged(You et al., 2017b). ARCT has been isolated in patients with hematologic malignancies, mainly with acute leukemia (de Carvalho Parahym et al., 2011; Chong et al., 2012; You et al., 2017b). A study analyzed the azole resistance mechanisms behind blood isolates of ARCT recovered from a neutropenic patient with acute lymphoblastic leukemia under posaconazole prophylaxis. ERG11p Y132F and S154C mutations and *MDR1* upregulation were related to azole resistance in these strains (You et al., 2017b). A study from Singapore also reported azole and echinocandin resistance in neutropenic patients with *C. tropicalis* fungemia (Chew et al., 2019a). Of note, the three isolates carried different FKS1p mutations, including F641L, D648V, and S645P (Chew et al., 2019a). Invasive devices are niches for biofilm production and resistance development. In China, a patient had a pan-echinocandin-resistant isolated from a thoracic drain after 18 days of exposure to micafungin. The isolate had a FKS1p mutation S80P(Xiao et al., 2018a). In Kuwait, a patient under mechanical ventilation had two echinocandin-resistant *C. tropicalis* recovered from endotracheal aspirates after short-term caspofungin exposure (Khan et al., 2018a).

*C. tropicalis* can be obtained from the microbiota of different wild and domesticated animal species, and livestock (Brito et al., 2009; Cordeiro et al., 2015; Zhai et al., 2021). In that context, fluconazole-resistant *C. tropicalis* has been recovered from mammals, birds, and crustaceans (Lord et al., 2010; Cordeiro et al., 2015; Álvarez-Pérez et al., 2016a; Ohno et al., 2019). A well-investigated case of *C. tropicalis* recurrent urinary tract in a dog showed a progressive increase in azole resistance after fluconazole exposure (Álvarez-Pérez et al., 2016a). No ERG11p mutations were detected despite high fluconazole MICs (>16mg/L), but isolates recovered after bladder irrigation with amphotericin B had an insertion at the *ERG3* gene of two consecutive guanine nucleotides resulting in a change in the reading frame (Álvarez-Pérez et al., 2016a). Captive dolphins may develop pulmonary mycosis which is usually treated with itraconazole and voriconazole (Delaney et al., 2013). Antifungal exposed dolphins have azole-resistant *C. tropicalis* recovered from the respiratory in Japan (Takahashi et al., 2010; Ohno et al., 2019). Moreover, one study showed that the water from the dolphins’ aquarium had been contaminated with azole-resistant *C. tropicalis* (Takahashi et al., 2010).

Azoles have been used in agriculture, aquaculture, and wood preservation (Jørgensen and Heick, 2021). Almost 10% and 15% of azoles sold in the world are being used in rice and soybean cultures, respectively (Jørgensen and Heick, 2021). Moreover, fluconazole is the most consumed antifungal in medicine and now is among the most common drugs found in the wastewater from hospitals(Cai et al., 2021). In China, it has been estimated that over 17 tons of fluconazole is discarded every year in the wastewater (Liu et al., 2017). Water treatment methods are not able to eliminate fluconazole that has poor biodegradability (Cai et al., 2021), and has been found in some countries in drinkable water (Reis et al., 2019; Assress et al., 2020). Therefore, azole fungicides are now widely found in nature and may be exerting selective pressure in ubiquitous *C. tropicalis*. In this context, recent evidence from Asia is pointing out that environmental and human ARCT isolates have identical genetic profiles, suggesting that the environment may be the source of these opportunistic resistant pathogens (**Table 2**). In Taiwan, ARCT clinical isolates belong mainly to close-related diploid sequence types (DSTs) that form a cluster named clonal complex 3 (CC3)(Chen et al., 2019). The DST225 from the CC3 has been reported from fruits and hospitals in Taiwan(Lo et al., 2017). In Wuhan, continental China, one study showed similar findings and demonstrated that human ARCP strains belong to close-related DSTs (**Table 2**). Like the previously described data from Taiwan, the DST225 was also found in the environment in Wuhan (Wang et al., 2020). Moreover, in Thailand, clinical ARCP isolates belonging to the close-related DST225 and DST506 have also been described (Tulyaprawat et al., 2020; Boonsilp et al., 2021).

## Conclusions

This study summarized the evidence showing that *C. tropicalis* is a relevant pathogenic species not only for humans but also for different domesticated and wild animals. Azole resistance in humans is particularly relevant in Asian countries but is emerging in other countries such as Turkey, Spain, and Algeria. Data from Brazil and India show that different animals may carry ARCT, but more information from other countries is necessary to better understand this phenomenon. Antifungal resistance mechanisms such as ERG11 mutations and efflux pumps hyperexpression have been described in ARCT clinical isolates, but veterinary and environmental molecular data are scarce. Azole fungicides are now widely found in nature and may be exerting selective pressure in ubiquitous *C. tropicalis*. As a consequence, environmental samplings have revealed a high prevalence of ARCT in the soil, freshwater, and seawater, especially in China and Brazil. Worrisome molecular data from China show clustering of ARCT from humans and the environment, highlighting the possible environmental source of antifungal resistance in the area.

## Author contributions

JJ and RL designed the manuscript. RL, FR, and JJ did a literature review. RL, FR, AC, and JJ wrote the manuscript. JJ and AC revised the manuscript. All authors contributed to the article and approved the submitted version.

## Funding

This study was supported by Fundação de Amparo à Pesquisa do Estado de São Paulo (FAPESP): 2017/02203-7. JNAJ has received a scholarship grant (FAPESP 2018/19347-4) and ALC a research Grant (FAPESP 2017/02203-7).

## Conflict of interest

The authors declare that the research was conducted in the absence of any commercial or financial relationships that could be construed as a potential conflict of interest.

## Publisher’s note

All claims expressed in this article are solely those of the authors and do not necessarily represent those of their affiliated organizations, or those of the publisher, the editors and the reviewers. Any product that may be evaluated in this article, or claim that may be made by its manufacturer, is not guaranteed or endorsed by the publisher.
